# Assessing the detection, reporting and investigation of adverse events in clinical trial protocols implemented in Cameroon: a documentary review of clinical trial protocols

**DOI:** 10.1186/s12910-015-0061-5

**Published:** 2015-09-29

**Authors:** Akoh Walter Ebile, Jerome Ateudjieu, Martin Ndinakie Yakum, Marceline Ngounoue Djuidje, Pierre Watcho

**Affiliations:** Department of Biomedical Sciences, University of Dschang, Dschang, Cameroon; Division of Health Operations Research, Ministry of Public Health, Yaoundé, Cameroon; M.A. SANTE (Meilleure Access aux soins de Santé), PO Box 33490, Yaoundé, Cameroon; Department of Biochemistry, Faculty of Science, University of Yaoundé I, Yaoundé, Cameroon; Ethics Committee for Research and Health in Central Africa, Yaoundé, Cameroun

**Keywords:** Detection, Reporting, Investigation, Adverse events, Clinical trial, Protocols, Cameroon

## Abstract

**Background:**

International guidelines recommend ethical and scientific quality standards for managing and reporting adverse events occurring during clinical trials to competent research ethics committees and regulatory authorities. The purpose of this study was to determine whether clinical trial protocols in Cameroon are developed in line with national requirements and international guidelines as far as detecting, reporting and investigating of adverse events is concerned.

**Methods:**

It was a documentary review of all approved clinical trial protocols that were submitted at the Cameroon National Ethics Committee for evaluation from 1997 through 2012. Data were extracted using a preconceived and validated grid. Protocol review process targeted the title, abstract, objectives, methodology, resources, and the chapter on safety.

**Results:**

In total, 106 (4.9 %) clinical trial protocols were identified from 2173 protocols seen in the archive and 104 (4.8 %) included for review. Seventy six (73.1 %) trials did not include the surveillance of adverse events as part of their objective. A total of 91 (87.5 %) protocols did not budget for adverse event surveillance, 76 (73.1 %) did not have a data safety management board (DSMB), 11(10.6 %) included insurance for participants, 47 (45.2 %) did not include a case definition for serious adverse events, 33 (31.7 %) described procedures to detect adverse events, 33 (31.7 %) described procedure for reporting and 22 (21.2 %) described procedure for investigating adverse events.

**Discussions:**

Most clinical trial protocols in Cameroon are developed to focus on benefits and pay little attention to harms. The development of national guidelines can improve the surveillance of adverse events in clinical trial research conducted in Cameroon. Adverse events surveillance tools and a budget are critical for an adequate planning for adverse event surveillance when developing trial protocols.

**Conclusion:**

Clinical trial protocols submitted in the Cameroon National Ethics Committee do not adequately plan to assess adverse events in clinical trial protocols. In order to improve on the safety of participants and marketed drug, there is a need to develop national guidelines for clinical trials by the government, and to improve evaluation procedures and monitoring of ongoing trials by the ethics committee.

**Electronic supplementary material:**

The online version of this article (doi:10.1186/s12910-015-0061-5) contains supplementary material, which is available to authorized users.

## Background

Unless information on harms is available, it is impossible to make a balance between benefits and risk of an investigational product/intervention in a clinical/field trial study. Scientific and ethical evidence have shown that adverse events are not well accounted for during Clinical trials [1, 2]. Clinical trials general aim to test the efficacy and safety of the investigational products/intervention, but many investigators/sponsors usually focus on the efficacy primarily because of fear that a proper assessment of harms may cause more trouble and discredit, than the fame and glory associated with successful reporting of benefits [3]. This may affect the quality of the research [4, 5], resulting to the production of drugs that are less safe to populations that eventually need them.

International guidelines such as the International Conference of Harmonization on Good Clinical Practice (ICH-GCP), CONSORT extension for Harms(for reporting results of clinical trials), the WHO reporting guidelines and the WHO Strategy on Research for Health recommend ethical and scientific quality standards for designing, conducting, recording and reporting trials that involve the participation of human participants. Compliance with these standards provides public assurance that the rights, safety and well-being of trial subjects are protected in accordance with the Helsinki Declaration, and that the clinical trial data are credible [6–9]. They recommend researchers and sponsors to report all adverse events resulting from clinical/field trials to competent Research Ethics Committees and to Regulatory authorities. During the development of protocols for such trials, these recommendations have to be taken into consideration.

In Cameroon, the Pharmacovigilance unit is in charge of drug safety surveillance while the National ethics committee is involved in national regulatory authorities of drugs and vaccines. There is political awareness of pharmacovigilance and its role to ensure the safety of pharmaceutical products [10] and on the organization and functioning of the National ethics Committee for the safety of research participants [11, 12]. But National guidelines to regulate research involving human participants and clinical/field trials do not exist. Drugs are administered in health facilities during clinical trials and in some health programs, yet adverse event resulting from these trials are usually not reported to these two bodies as international guidelines recommend. This study was therefore conducted on clinical trial protocols to assess how much investigators and sponsors have planned to assess adverse events during clinical trials. That is, how much they have planned to collect data on adverse events, investigate them and discuss objectively whether or not the benefit outweighs the risk. This assessment was done by determining the proportion of clinical trials that included adverse events as part of the research objectives, outcomes and follow up variables. It also assessed the resources, methods and procedures that were used in the surveillance of adverse events. Findings of this study will help to identify intervention needs to ameliorate on the safety of research participants during clinical trials conducted in Cameroon. It will also provide evidence to decision making that will help to improve the respect of ethical and scientific standards of conducting clinical research and the practice of pharmacovigilance in Cameroon and other resource limited countries.

## Methods

This study was issued ethical clearance by the Cameroon National Ethics Review Committee (CNERSH) with reference number 2013/11/385/L/CNERSH/SP. A confidentiality agreement was signed before access to the protocols was granted. Personal information about the investigators or sponsors was not collected. Data collection was anonymous.

It was a documentary review (cross-sectional study) of approved clinical trial protocols that had been submitted to the Cameroon National Ethics Committee for evaluation from 1997 through 2012 inclusive. All hard copies of eligible clinical trial protocols were reviewed line by line and data was extracted on a grid.

The Cameroon National Ethics Review Committee is an independent body, from all political, institutional, professional and economic influence. It is one of the major actors involved in health research in Cameroon. Its main role is to safe-guard the dignity, the rights, the security, the physical integrity and the well-being of potential participants in research projects conducted in Cameroon. It also ensures collaboration and net-working with other ethics review boards at the national and international levels. It is involved in the national regulatory authority of drugs and vaccines in Cameroon. It was created in October 1997 by the ministerial order [11]. Since then, it has been carrying out its activities. This study was conducted in 2013, involving the review of clinical trial protocols from 1997 through the year 2012 inclusive.

It was an exhaustive study. All clinical trial protocols that were submitted for evaluation at the CNERSH since its creation in 1997 were targeted. Approved protocols that were being implemented or that were to be implemented were eligible for review and were included in the study while unapproved protocols and protocols that were still under review were excluded (See Additional file 1: Operatioanl Definitions used).

All protocols received by the CNERSH are eventually archived both as hard and electronic copies. In the physical archive system of hard copies, clinical trial protocols are not separated from other research protocols involving the participation of human subjects. It was therefore necessary to first identify and separate all clinical trial protocols from the others. To do this, all archived protocols from 1997 through 2012 were reviewed one after the other chronologically according to the way they were physically arranged in the archive system. The Title, abstract and objective of each protocol was reviewed line by line in order to identify protocols that were clinical trials. Once a clinical trial protocol was identified, it was attributed a code and kept aside for the next stage that involved a detail review for data extraction. Four persons were involved in this stage (two holders of a Master’s degree in Epidemiology and two Medical doctors). The CNERSH demands the submission of four hard copies of research protocols for evaluation. Thus the four copies of a research protocol were reviewed by all four persons of the team simultaneously. When there were disagreements about the decision to include a protocol as a clinical trial or not, reference was made to the protocol and to standard definitions before decision taking. Once all clinical trial protocols were identified, they were reviewed one after the other, in succession, and depending on their identification codes, for data extraction. Two persons who were holders of masters in Epidemiology were involved at this stage. They simultaneously reviewed each clinical trial protocol for data extraction. When there was disagreement, reference was made to the protocol and to international guidelines before decision taking.

In each clinical trial protocol, the following sections were reviewed for data extraction; the cover page, abstract, objectives, study design, methodology, implementation procedure, availability of insurance, budget, the chapter on safety and the availability of a Data Safety Management Board (DSMB). Each of the sections mentioned above were reviewed line by line to identify parameters of interest. Data collected from each protocol was noted on a structured grid.

Regarding the availability of resources, protocols were reviewed to see if there was a budget allocation for adverse event surveillance, and if the budget incorporated training on adverse event surveillance. The availability of insurance welfare for research participants and the inclusion of a role to be played by the data management safety board (DSMB) were also verified.

Regarding procedures or methods of adverse event surveillance, parameters of interest included; the mode of detection of adverse events, availability of persons assigned to detect, and the existence of clear case definitions of serious and minor adverse events. The procedures or methods of adverse event reporting was assessed with indicators that verified the availability of persons to report, the reporting target for both expected and unexpected adverse events, existence of a reporting time frame, and reporting procedures. Assessment of procedures or methods of adverse event investigation were based on indicators that evaluated the procedures of investigation, existence of stopping guidelines, existence of a plan to analyze data on adverse events and the existence of plan to manage serious adverse events.

At the end of each working day, data collected by the two data collectors were compared for differences. In case there was a difference, reference was made to the original protocol and to existing guidelines if need be and a resolution was taken. Protocols that were in foreign language (non-French or English) were reviewed with the help of translators. Three translators were used; they were holders of a master’s degree in the language involved. The languages were German, Spanish and Italian.

The data entry screen was created in Epi Info. There was double data entry; that is the same data was entered by two different persons, after which the databases were compared for discrepancies and resolved using the utility in Epi info labeled “Data compare”. All proportions were calculated at 95 % confidence interval.

## Results

**Fig. 1:**
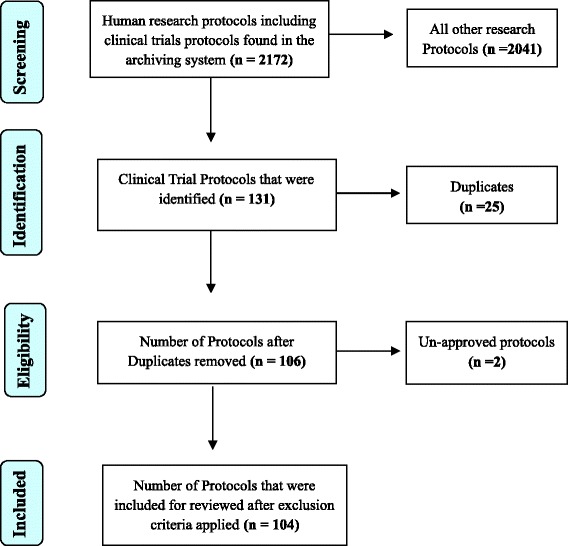
Flow diagram to describe the procedures of identification and inclusion of clinical trial protocols for review

**Fig. 2:**
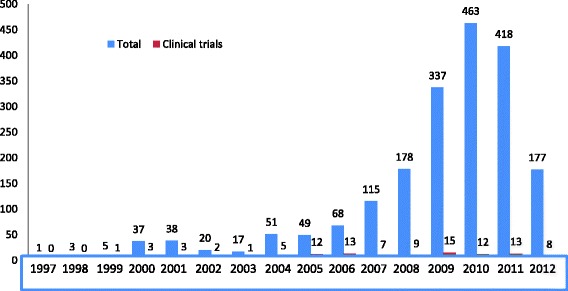
Evolution in the number of clinical trials conducted in Cameroon

A total of 2172 research protocols involving the participation of human subjects were seen in the archive. Among these, 106 (4.9 %) were clinical trial protocols. In total, 104 (4.8 %) were reviewed for data extraction. We note that 31 (29.8 %) were locally sponsored trials while 71 (68.3 %) were internationally sponsored trials. The Prisma flow diagram in Fig. 1 describes the procedure of identification and selection of eligible clinical trial protocols for review. We also note from Fig. 2 that the rate of conducting clinical trials in Cameroon is on an increase. The highest number of clinical trials conducted was between 2005 and 2011.

### Adverse events as part of study objectives/outcome and the availability of resources

We observe that 28 (26.9 %) protocols had at least one objective that incorporated the assessment of adverse events, 18 (17.3 %) had at least one adverse event as part of the study outcome and 66 (63.5 %) included adverse event among the variables to be followed-up.

**Fig. 3:**
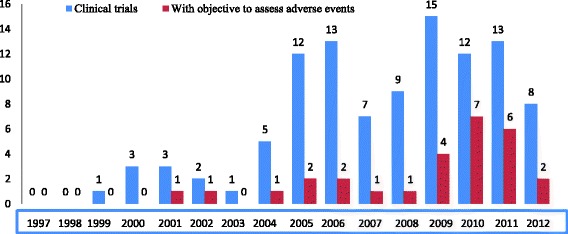
Evolution of the number of protocols that include the assessment of adverse events as part of the objectives of the trials

In Fig. 3, we observe that the proportion of clinical trial protocols that include the assessment of adverse event as part of the study objectives shows an improvement with time. Regarding the availability of resources, 13 (12.5 %) planned a budget for adverse event surveillance. Among these, none had included budget for training on adverse event surveillance. Fourteen (13.5 %) included adverse events in the sample size calculation, 11 (10.6 %) planned for a post-trial insurance welfare for research participants, 28 (26.9 %) had a Data Safety Management Board (DSMB) and 12 (11.5 %) had an investigator brochure.

### Procedures of adverse event detection

We observed that 31 (29.8 %) planned to use an active surveillance technic to detect adverse events while 10 (9.6 %) planned to use passive surveillance. Also, 45 (43.3 %) did not specify the technique they would use to detect adverse events while 15 (14.4 %) planned to use a mixed (active and passive) technique to detect adverse events. Table 1 shows some parameters needed in the surveillance of adverse events and that were assessed, stratified by sponsors. We observe that in general, internationally sponsored trials provide more parameters than locally sponsored trials.

**Table 1:** Description of parameters regarding the detection of adverse events

		National sponsors	International sponsors	
Indicator	Observation	Yes (%)	Yes (%)	Total (%)
Persons assigned to detect adverse events	104	4 (3.8)	37 (35.6)	41 (39.4)
Defined a minor adverse event	104	4 (3.8)	53 (51.0)	57 (54.8)
Defined a serious adverse event	104	4 (3.8)	53 (51.0)	57 (54.8)
Indicated a time frame to detect adverse events	104	3 (2.9)	41 (39.4)	44 (42.3)
Described a Procedure for detecting adverse events	104	2 (1.9)	31 (29.8)	33 (31.7)
Case Report Form	96	4 (3.8)	35 (33.7)	39 (37.5)

### Adverse events reporting procedures

In 36 (34.6 %) protocols, someone was assigned in charge of reporting adverse events. Twenty eight (26.9 %) indicated the investigator as the one in charge of reporting, 66 (47.1 %) did not specify persons in charge for reporting adverse events, 40 (38.5 %) specified the time limit during which any detected serious adverse event must be reported to the appropriate body, and 33 (31.7 %) had a clearly described procedure to report serious adverse events. Table 2 shows the distribution of the specified time frame for reporting adverse events as identified in the reviewed clinical trial protocols by sponsors. We observed that internationally sponsored trials adhere to recommendations better than locally sponsored trials.

**Table 2:** Description of reporting targets of suspected Adverse Events by type of sponsors

	National sponsors	International sponsors
When to report adverse events	Frequency	Frequency
Serious adverse events		
No later than 1 hour	0	0
No later than 24 hours	0	31
No later than 7 days	1	2
No later than 15 days	0	1
Others	0	5
Did not specify	30	32
Total	31	71
Unexpected adverse events		
No later than 1 hour	0	1
No later than 24 hours	0	12
No later than 7 days	1	1
No later than 15 days	0	3
Others	0	6
Did not specify	30	48
Total	31	71

### Adverse events investigation procedure

Among the 104 reviewed protocols, 23 (22.1 %) planned to manage serious adverse events, 6 (5.8 %) planned to manage unexpected serious adverse events, and 22 (21.2 %) described a procedure to investigate any adverse event that will be detected in the course of the trial. Thirty five (33.7 %) had a stopping guideline or conditions to stop the trial for serious adverse events and 24 (23.1 %) described a plan to analyze adverse event data.

## Discussions

This study intended to determine how much researchers have planned to assess adverse events in clinical trial studies conducted in Cameroon. This evaluation was done through the review of approved clinical trial protocols at the Cameroon National Ethics Committee. Protocols need to be evaluated by research ethics committees (RECs) to assess whether all scientific, ethical, and legal requirements for conducting research with human subjects are met [13, 14]. The key findings of this study indicated that 26.9 % of clinical trials conducted in Cameroon included the surveillance of adverse events as part of the objective. Similarly, 17.3 % included adverse events as part of the study outcome under investigation. Regarding adverse event surveillance procedures, 31.7 % described a procedure for detecting, 31.7 % for reporting and 21.2 % described a procedure to investigate adverse events. It was noted that 12.5 % of the protocols included a budget for adverse event surveillance. Also, 13.5 % included adverse events in the sample size calculation and 10.6 % planned for participant’s insurance care. Few trials (28.9 %) indicated a role to be played by the DSMB, 33.7 % had a stopping guideline and 41.7 % had a case report form.

Over the past two decades, we observe a steady increase in the number of clinical trials conducted in Cameroon until 2012 where we see a sudden decrease. A possible explanation to the sudden drop in 2012 may be due to the fact that the ministerial decision in April 2012, resulted to the restructured, organization and functioning of the CNERSH [12]. After which its office that was initially located in Bastos adjacent to Lycee le Nkoleton since it was created was then transferred to the Ministry of Public Health. We can also estimate from the results of this study that clinical trials make up about 5 % of all research conducted on human subjects in Cameroon. We also noted that the number of clinical trials conducted in Cameroon is significantly different from that recorded in the WHO Clinical trial Registry. A study published in 2011 through a simple search by location on the clinical trial website https://clinicaltrials.gov/ revealed 24 Clinical trials in Cameroon. On the WHO International clinical trial Registry Platform (ICTRP), the numbers were similar whereas in this study, by that same year (2011), we identified 99 clinical trial protocols. This indicates that many clinical trials that are conducted in Cameroon are not registered in the clinical trial registry. This study did not explore the reasons for the low registration rate of clinical trials being conducted in Cameroon though we believe that it may be due to lack of awareness or motivation.

The International Conference of Harmonization on Good Clinical Practice (ICH-GCP),the WHO guidelines, the WHO Strategy on Research for Health, and the Consolidated Standard for Reporting Trials (CONSORT) extension on Harms recommend ethical and scientific standards for conducting, and reporting results of clinical trials. Clinical trials need to be developed carefully to give a fair and balance report between risks and benefits, wherever the research is conducted [15–17]. Clinical trials have a goal to assess the safety and efficacy of the product/intervention under investigation [6]. In order to say that the product is safe and effective for human use, a fair balance assessment of both the efficacy and safety must be made and the benefits must outweigh the risk. It is therefore impossible to make this balance when data/information on either of safety and efficacy is lacking. In this study, we observed that most protocols did not include the assessment of adverse events as part of the study objectives and outcomes. This is an indication that most of the trials were focusing on the benefits, confirming the findings of a study that showed that less attention is given to assess harms than benefits during clinical trials [1, 2]. Though we did not explore factors that are contributing to this, we think that the lack of budget, limitation in knowledge of researchers in conducting research in accordance with the methods and procedures of good clinical practice or the fear that a proper assessment of harms may cause discredit of the research findings could be some of the reasons. Similar studies have shown this [3].

Adverse events surveillance needs to be considered when budgeting for clinical trials. The detection of adverse events during clinical trials requires that the sample size be large enough to increase the statistical power to detect adverse events. It is therefore vital to consider adverse events when calculating the sample size so that it will be large enough to be able to detect adverse events. A large sample size will definitely require an increase in budget as well. This study has indicated that few clinical trials budgeted to survey adverse events. That can be proven by the fact that only 14 (14.9 %) of the trials included adverse events in the sample size calculation. This is evidence that little attention is given to assess the safety of the products under investigation.

In Cameroon, one of the key requirements for obtaining administrative clearance for clinical trials involving human subject participants is a signed insurance document [10]. This document engages the investigators and sponsors to manage all cases of adverse events, both short term or long term that may occur during the trial. This study has shown that globally, more than 90.4 % of the trials did not plan any insurance for participants. This implies that participants of such studies will be liable to both physical damage (such as pains, incapacities and even deaths) and economic consequences (direct and indirect cost of treatments and rehabilitation) all by themselves in case they suffer from an adverse event.

Looking at this, pre and post the arête of 2009, only 2 % of the protocols planned insurance for participants before 2009. This low proportion can be explained by the fact that there was no decision regulate the conduct of clinical trials. However, after 2009, the situation that was devastating (2 %) now improved to about 10.6 %. We expected this proportion to be higher, this can be explained by the fact that many of the reviewed protocols were field trials and other types of intervention trials studies that did not necessarily required the insurance of participants. We can thus say that the adherence to national requirements and to international guidelines concerning the insurance of research participants during clinical trials in Cameroon is improving though a lot still needs to be done to become standard.

Adverse event surveillance during clinical trials involves the detection, reporting to the appropriate body (Sponsor or regulatory authorities) within acceptable time limits depending on the seriousness of the event and the investigation of adverse events to determine a causality relationship with the substance being investigated. These procedures have to be clearly described in clinical trial protocols and respected whatever the type or phase of the clinical trial concerned. This study found out that these procedures are not well elaborated in clinical trial protocols to be implemented in Cameroon. According to the ICH GCP and WHO guidelines for good clinical practice, these procedures should include specifications such as who should detect, when to detect, how to detect, when to report, where to report, how to report and how to investigate any detected adverse events during the trial. We noted that most of the protocols did not specify those important elements. For example, 60.6 % did specify who detects adverse events during the trial and 52.9 % specified who should report adverse events. This confirms the findings of a studies that have revealed that in most clinical trials (especially large trials), adverse events is usually self-reported and is not independently verified [18]. Other studies have also shown that there is a relative poor agreement between self-reports and the active detection of adverse events [19]. In the same trend, most did not state the time limits of reporting based on the seriousness of the adverse events. For example, serious adverse events ought to be reported immediately and not later than 24 h to the sponsors and to ethics review committees. Investigating adverse events is vital to establish a causality relationship between the investigational product and the adverse event, yet most of the protocols said nothing about this. All these is evidence that adverse events are not well accounted for during clinical trials in Cameroon just as in most other settings [17]. From Tables 1, 2 and 3, we clearly observe that most of the trials that did not sufficiently plan to survey adverse events were locally sponsored trials. This is due to the fact that national guidelines to regulate the conduct of clinical trials do not exist. It may also be due to lack of knowledge or awareness of most local investigators/sponsors of clinical trials in Cameroon.

**Table 3:** Description of reporting targets of adverse events by sponsors

	National sponsors	International sponsors
Target to report adverse events		
Expected adverse events		
Investigator	1	5
Sponsor	1	13
DSMB	0	1
Ethics Board	1	4
Not specified	28	43
Total	31	71
Unexpected adverse events		
Investigator	0	2
Sponsor	0	7
DSMB	0	1
Ethics Board	1	3
Others	1	10
Not specified	29	50
Total	31	71

The DSMB is vital in clinical trial. It assesses at intervals the progress of a trial, the safety data, and the critical efficacy endpoints. It recommends to the sponsor whether to continue, modify, or stop the trial based on their assessment of the data. They have a well described operational procedure and a stopping guideline. This study found out that most of the trials did not have a DSMB and a stopping guideline. The stopping guideline provides evidence to the decision to stop or continue the trial depending on safety and efficacy results. This confirms the findings of studies that revealed that the number of clinical trials that have been stopped earlier has increased significantly over the past 15 years and that most of them failed to report at least one of several key details about the decision to stop the trial [20, 21]. For example, it was also shown that prematurely stopped Randomized Controlled Trials(RCT) often fail to adequately report relevant information about the decision to stop early [22]. We also observed that many of the protocols did not have a case report form. The Case Report Form (CRF) is a useful tool for collecting information on adverse events during clinical trials. This form has to include in the submitted protocol file to be evaluated. The fact that most of the trials did not include this form is an indication that they were not ready to assess adverse events.

Some limitations of this study includes the fact that only protocols in the national ethics committee were reviewed and there were certainly some protocols that were not submitted for ethical approval. Secondly, data collection depended on the capacity of the data collector to read, understand and collect the right information, indicating that there might have been few issues of data quality. But this was minimized firstly by selecting persons who have acceptable knowledge on the subject ie holders of master’s degree in Epidemiology and Medical doctors, furthermore, they were trained on data collection process. We did not visit sites where clinical trials are being conducted to verify these findings. We therefore recommend a more in-depth study to assess ongoing studies to confirm these findings.

## Conclusion

Clinical trials protocols submitted at the Cameroon National Ethics Committee do not adequately plan to assess adverse events in clinical trial protocols. Researchers and sponsors of clinical trials in Cameroon do not adhere to national requirements and international norms and standards when developing clinical trial protocols. Therefore, the procedures of detecting and managing adverse events needs to be improved in clinical trial research protocols submitted to the National Ethics Committee. In order to improve this, we recommend the following:

1. Development of national guidelines to regulate the conduct of clinical trials;
2. Make amendments to the standard operating procedures for evaluating clinical trial protocols to include parameters for the surveillance of adverse events;
3. The use of standardize definitions of adverse events and serious adverse events by researchers and local and international authorities;
4. Providing guidelines to actors involved (researchers, sponsors *etc.*), training actors on how to use guidelines through seminars and workshops,
5. Encourage actors involved to important resources sites such as the EQUATOR Network guidelines,
6. Promote the adherence to norms and guidelines through periodic monitoring of ongoing trials
7. Considering adverse events surveillance when making a budget.
8. We also recommend that the national ethics committee should reinforce the capacity of its members in clinical trial and pharmacovigilance and reject protocols that do not sufficiently respect the rights, safety and dignity of research participants.
9. We equally encourage a strong collaboration between the national pharmacovigilance center and the national ethics committee since they are the two structures involved in drug safety surveillance and national regulatory authority of drugs and vaccines in Cameroon respectively;
10. Encourage researchers/sponsors to register clinical trial research protocols and that the National Ethics Committees should collaborate with registration centers to ensure that all approved protocols are registered;
11. This study should be replicated in other countries;
12. A follow up study should be conducted in 5 years to see its evolution.

## Additional file

Additional file 1:
**Operational definitions used in the research.** (DOCX 14 kb)

## Abbreviations

ICH: International Conference on Harmonization
CRF: Case Report Form
GCP: Good Clinical Practice
DSMB: Data Safety Management Board
CNERSH: Cameroon National Ethics Review Committee
