# Supplementary material for: Assessing the detection, reporting and investigation of adverse events in clinical trial protocols implemented in Cameroon: a documentary review of clinical trial protocols
Source: BMC Med Ethics. 2015 Sep 29;16:67. doi: 10.1186/s12910-015-0061-5 (PMC4589194; doi:10.1186/s12910-015-0061-5)
Supplement: Additional file 1: — Operational definitions used in the research. (DOCX 14 kb) [file 12910_2015_61_MOESM1_ESM.docx]

**Operational Definitions Used in the Research**

The following operational definitions were considered during data collection:

***Clinical Trial Protocol:*** A document that describes the objective(s), design, methodology, statistical considerations, and organization of a trial (ICH Definition).

***Clinical Trial:*** Any research study that prospectively assigns human participants or groups of humans to one or more health-related interventions to evaluate the effects on health outcomes. Interventions include but are not restricted to drugs, cells and other biological products, surgical procedures, radiological procedures, devices, behavioral treatments, process-of-care changes, preventive care, etc. (WHO definition).

***Adverse Events:*** Any untoward medical occurrence in a patient or clinical investigation subject administered a pharmaceutical product and which does not necessarily have a causal relationship with this treatment (ICH Definition).

***Serious Adverse Event:*** Any untoward medical occurrence that at any dose, can result to death, is life-threatening, requires inpatients hospitalization or prolongation of hospitalization, results in significant disability or birth defect (ICH Definition). .
